# Heart Rate Variability as an Index of Differential Brain Dynamics at Rest and After Acute Stress Induction

**DOI:** 10.3389/fnins.2020.00645

**Published:** 2020-07-02

**Authors:** Tara Chand, Meng Li, Hamidreza Jamalabadi, Gerd Wagner, Anton Lord, Sarah Alizadeh, Lena V. Danyeli, Luisa Herrmann, Martin Walter, Zumrut D. Sen

**Affiliations:** ^1^Department of Psychiatry and Psychotherapy, University of Tübingen, Tübingen, Germany; ^2^Department of Psychiatry and Psychotherapy, Jena University Hospital, Jena, Germany; ^3^Max Planck Institute for Biological Cybernetics, Tübingen, Germany; ^4^Clinical Affective Neuroimaging Laboratory (CANLAB), Magdeburg, Germany; ^5^QIMR Berghofer Medical Research Institute, Brisbane, QLD, Australia; ^6^Leibniz Institute for Neurobiology, Magdeburg, Germany

**Keywords:** heart rate variability, resting-state fMRI, dynamic functional connectivity, heart-brain interaction, stress

## Abstract

The brain continuously receives input from the internal and external environment. Using this information, the brain exerts its influence on both itself and the body to facilitate an appropriate response. The dynamic interplay between the brain and the heart and how external conditions modulate this relationship deserves attention. In high-stress situations, synchrony between various brain regions such as the prefrontal cortex and the heart may alter. This flexibility is believed to facilitate transitions between functional states related to cognitive, emotional, and especially autonomic activity. This study examined the dynamic temporal functional association of heart rate variability (HRV) with the interaction between three main canonical brain networks in 38 healthy male subjects at rest and directly after a psychosocial stress task. A sliding window approach was used to estimate the functional connectivity (FC) among the salience network (SN), central executive network (CEN), and default mode network (DMN) in 60-s windows on time series of blood-oxygen-level dependent (BOLD) signal. FC between brain networks was calculated by Pearson correlation. A multilevel linear mixed model was conducted to examine the window-by-window association between the root mean square of successive differences between normal heartbeats (RMSSD) and FC of network-pairs across sessions. Our findings showed that the minute-by-minute correlation between the FC and RMSSD was significantly stronger between DMN and CEN than for SN and CEN in the baseline session [*b* = 4.36, *t*(5025) = 3.20, *p* = 0.006]. Additionally, this differential relationship between network pairs and RMSSD disappeared after the stress task; FC between DMN and CEN showed a weaker correlation with RMSSD in comparison to baseline [*b* = −3.35, *t*(5025) = −3.47, *p* = 0.006]. These results suggest a dynamic functional interplay between HRV and the functional association between brain networks that varies depending on the needs created by changing conditions.

## Introduction

The body and the brain are interconnected by dynamic structural and functional networks. These networks provide multi-level interactions and allow conscious and subconscious reactions to constantly changing environmental conditions (McCraty et al., 2009; Smith et al., 2017). Network physiology proposes a new framework to understand the coordination and information integration across different organ systems that give rise to various physiologic states at organism level (Ivanov et al., 2016). Interactions between the brain regions, organs, and organ systems vary dynamically, allowing the same network structure or subsections of the network to be associated with many physiological and psychological states (Honey et al., 2007; Bashan et al., 2012; Bartsch et al., 2015). Recent research on the dynamic temporal interaction between the heart and the brain enriched our understanding beyond the known anatomical connections and physiological regulations (Bashan et al., 2012; Chang et al., 2013; Bartsch et al., 2015; Faes et al., 2016; Young et al., 2017).

Interaction between the cardiovascular system and central nervous system (CNS) is facilitated through reflex arches and the modulatory action of the cortical networks upon them. The central autonomic network (CAN; Benarroch, 1993) refers to a functional unit of brain areas that modulate the autonomic activity depending on the organism’s current and expected needs. This network includes brainstem regions, such as dorsal vagal motor nucleus or nucleus of the solitary tract, higher subcortical regions (i.e., hypothalamus and amygdala), and cortical regions [i.e., anterior cingulate cortex (ACC), insula, and medial prefrontal cortex (mPFC)], and modulates the balance between the activity of sympathetic and parasympathetic systems (Benarroch, 1993; Beissner et al., 2013; Dampney, 2015; Shoemaker and Goswami, 2015). Concurrent analyses of heart rate (HR) or heart rate variability (HRV) and blood-oxygen-level dependent (BOLD) signal at rest or during an emotional, cognitive, or motor task indicated that some additional cortical regions, such as dorsolateral PFC (dlPFC), exert influence on HRV in coordination with CAN, although they are not closely connected to the autonomic centers in the brain stem (Napadow et al., 2008; Smith et al., 2014; Young et al., 2017). HRV corresponds to the variation in the time interval between two successive R waves and has been associated with the various cognitive (Hansen et al., 2009; Dalise et al., 2020) and affective functions (Melzig et al., 2009; Miller et al., 2019). Thayer and Lane (2000) put forward the neurovisceral integration (NVI) model centered on CAN regions and provided a framework for understanding the integration of cognitive, affective, and autonomic information. HRV was proposed as an index of the degree to which flexible and adaptive interaction between the human organism and the environment can be achieved (Thayer and Lane, 2000, 2009; Thayer and Brosschot, 2005; Thayer et al., 2012).

Smith et al. (2017) extended the NVI model and described the roles in efferent and afferent information processing of each brain region constituting the CAN. The lower levels of hierarchy correspond to the information processing at the level of the brainstem nuclei and subcortical regions that integrate upcoming information from different bodily sources (Smith et al., 2017). On the other hand, the core neural networks, such as the Default Mode Network (DMN) and the Salience Network (SN), constitute the higher levels of hierarchy, where information in terms of exteroception, interoception, and memory is integrated by taking into consideration not only the present, but also the expected future metabolic needs, which are related to long-term goals. The Central Executive Network (CEN) processes goal-relevant information within a circuit of highly connected hub regions that constitute SN and DMN (Dehaene, 2014; Barrett, 2016; Smith et al., 2017). The synchronous activity of these networks may provide a “global workspace” that allows the emergence of conscious representations that are significant to the goals or overall state of the organism (Zylberberg et al., 2010, 2011; Dehaene and Sigman, 2012). These three core brain networks, namely CEN, SN, and DMN, have been identified by functional connectivity (FC) analyses predominantly at resting state fMRI, while subjects lie in the scanner and are not asked to engage in any particular task. Since the ascending inputs from the visceral organs continuously reach numerous cortical and subcortical regions, many researchers claim that the visceral signals are the continuous internal stimuli that contribute and shape the spontaneous brain activity and intrinsic brain-networks (Azzalini et al., 2019; Kim et al., 2019).

If HRV is an index of the flexible interaction between the heart and the brain, the relationship between HRV and FC patterns in the brain networks should vary across different psychological and physiological states. Stress is a perturbed state at the whole-body level induced by extrinsic or intrinsic stimuli (Oken et al., 2015). Stress induction activates limbic regions such as amygdala, which is also under influence of cortical regions such as vmPFC; consequently, hypothalamic-pituitary-adrenal axis and sympathetic nervous system are activated, leading to increased cortisol levels and heart rate (Tafet and Nemeroff, 2016). HRV as measured by the root mean square of successive differences between normal heartbeats (RMSSD) or high frequency (HF) power are suppressed as a reaction to acute stress induction (Kim et al., 2018; Castaldo et al., 2019). Three dominant brain networks, namely DMN, CEN, and SN, are known to be modulated by acute stress (Hermans et al., 2011; Vaisvaser et al., 2013; Maron-Katz et al., 2016; van Oort et al., 2017). For example, during acute stress induction by affective stimuli, the activation and functional connectivity of SN as well as the activation in DMN regions increase, while the activation in CEN remains unchanged (van Oort et al., 2017). On the other hand, less consistent findings were reported during acute stress induction by psychosocial stress tasks, while the role of brain regions being part of CEN, SN, and DMN is consistently reported during stress induction (van Oort et al., 2017). Previous rs-fMRI studies indicated a carry-over effect of acute psychosocial stress induction on these three intrinsic brain-networks (Vaisvaser et al., 2013; Maron-Katz et al., 2016). An increased functional connectivity between brain regions of the SN and DMN was associated with the subjective stress levels (Vaisvaser et al., 2013; Quaedflieg et al., 2015; Maron-Katz et al., 2016). However, whether the association of HRV with the FC between these three networks changes before and after stress induction remains to be tested.

More recent views of resting-state FC (rsFC) integrate both static and dynamic components: the static component represents stable dimensions of overall FC during the whole fMRI session, and the dynamic component represents the processes by which networks and subnetworks unite and dissolve over time (Chang and Glover, 2010; Handwerker et al., 2012; Hutchison et al., 2013; Kaiser et al., 2016). Indeed, dynamic FC (dFC) was shown to co-fluctuate with HRV and arousal (Chang et al., 2013; Young et al., 2017). By using a sliding window approach to calculate FC maps and HRV, Chang et al. (2013) showed that FC between dorsal ACC and precuneus was significantly associated with the HRV. However, the association of HRV with the dynamic and static FC between the three core brain networks remains to be elucidated.

Here, we investigated the temporal association between RMSSD and FC between the three core brain networks – DMN, SN, and CEN – at two different resting-state scan sessions: baseline and just after a psychosocial stress task. In this study, we hypothesized that RMSSD correlates with the FC between the three network pairs (DMN-SN, DMN-CEN, and SN-CEN) at rest and these patterns are altered by acute stress. Minute-by-minute associations were assessed by the multilevel analysis instead of the common summary-statistics model (Chang et al., 2013; Vaisvaser et al., 2013; Young et al., 2017), in which first-level (e.g., subject or time window) data are aggregated on group level and the variance of first-level data cannot be taken into account. Moreover, this is the first study to examine how the functional association of HRV with FC between the core brain networks change after acute stress induction.

## Materials and Methods

### Sample

This study was part of a randomized, placebo-controlled, double-blind, two-period crossover clinical trial (ClinicalTrials.gov Identifier: NCT02602275). The Ethics Committee of the Medical Faculty of the University of Magdeburg approved the experimental protocol of the study and the study was conducted in accordance with the Declaration of Helsinki (World Medical Association, 2002). Participants provided written informed consent prior to participation and received financial compensation for their participation. Participants were screened for MR compatibility and medical and psychiatric examination, including a Structured Clinical Interview for DSM-IV Axis I, (SCID) was performed. During the screening visit, the stress level was assessed by means of the Perceived Stress Scale (PSS) and Trier Inventory for Chronic Stress – Screening Scale for Chronic Stress (TICS-SSCS). Subjects with low (PSS score = 9, TICS-SSCS score < 9) and high (TICS-SSCS score > 36) levels of chronic stress were not included in the study to ensure subject’s susceptibility to the stress and to avoid a ceiling effect of the stress sensitivity. Subjects were excluded if they were diagnosed with any psychiatric or serious somatic disease or were not suitable for MRI scanning.

A group of 40 healthy male subjects aged 31–59 years was enrolled in the study. Since males and females showed distinct stress responses modulated by the difference in hormonal levels caused partly by menstrual cycle (Goldstein et al., 2010; Saladin et al., 2015), only male participants were recruited for this study to reduce the variability within stress responses. The sequence of placebo or active compound was randomized. After randomization, one subject was excluded because of an incidental finding during the baseline MR measurement; therefore, 39 subjects (age = 43.7 ± 9.8) were included in this study. Nineteen participants received a placebo and the remaining 20 participants received the *verum* on the first measurement day. On the second measurement day, after a wash out period (7–35 days after the first measurement day), treatment was crossed-over.

### Study Design

**FIGURE 1:**
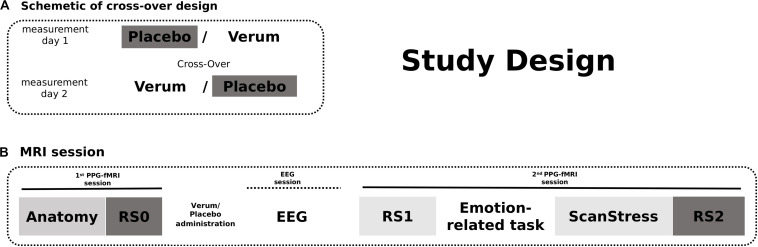
The study design. **(A)** Participants received either Verum or Placebo on two measurement days. The treatment was cross-overed. **(B)** On both measurement days, fMRI scans were acquired in two sessions. The first session began with an anatomical scan followed by a baseline resting-state measurement. After intake of the Placebo/Verum treatment, EEG paradigms were performed outside the scanner. The second MRI session included the shortened ScanStress task measurement following two other task measurements as well as two resting-state measurements before and after the tasks, respectively. In this publication, only the analyses of RS0 and RS2 fMRI scans on the day of placebo intake were reported.

On both measurement days, Days 1 and 2, after an anatomy scan and acquisition of 12-min baseline rs-fMRI (RS0), participants received either placebo or *verum* [a herbal medicinal product, Neurexan (Nx4)]. Subsequently, they performed two attention tasks with simultaneous EEG acquisition outside the scanner before entering another fMRI session. During that session, participants underwent a psychosocial stress paradigm, which was preceded by two emotion-related fMRI tasks reported in separate publications. A 12 min resting-state sequence was acquired before the fMRI tasks (RS1) as well as immediately after the psychosocial stress induction (RS2) (see Figure 1). Here in the current study, only RS0 (baseline) and RS2 (after stress induction) scans of the day of placebo intake were analyzed. RS1 scans were not included in the main analysis, but findings obtained from the analysis of all three scanning time points are reported in the Supplementary Results. The photoplethysmography (PPG) signal was continuously monitored during all scans. The PPG sensor was attached to the proximal phalanx of the left index finger.

To induce psychosocial stress, we used a shortened version of the ScanSTRESS task inside the scanner, which was previously shown to induce stress-related changes in the brain, including changes in physiological measures and hormone levels (Streit et al., 2014, 2017; Dimitrov et al., 2018). During the task, participants performed demanding arithmetic calculations and mental rotations under time pressure and with feedback regarding correctness and speed from a jury panel shown live on the screen (stress blocks). As a control condition, the questions of both types were easy and there was no time pressure or evaluative feedback (control blocks). In total, each block of 40 s (shortened version) was presented four times, with stress and control blocks on an alternating basis. In the middle of the experiment, participants received negative verbal feedback from the panel to increase the stress level. The detailed description of the task can be found in Streit et al. (2014).

The level of anxiety and nervousness was assessed using the State-Trait Anxiety Inventory (STAI-X1) and a continuous visual analog scale for nervousness (VAS-Nerv) at baseline as well as before and after stress induction. Saliva samples for alpha-amylase and cortisol measurements were collected at eight-time points before and after the stress task via the saliva collection device Salivette^®^ (Sarstedt, Germany) in addition to morning cortisol samples. Salivary cortisol and alpha-amylase levels were analyzed using commercial enzyme-linked immunosorbent assays according to the manufacturer’s instructions.

### MRI Acquisition

All data (structural and functional MRI) were acquired on a Philips 3T scanner in Magdeburg. First structural T1-weighted images were measured using a TFE sequence with the following parameters: 274 sagittal slices covering the whole brain, flip angle = 8°, 256 × 256 matrix, voxel size 0.7 × 0.7 × 0.7 mm^3^. The functional MRI data were acquired using following scanner settings: 34 axial slices covering the whole brain, TR = 2,000 ms, TE = 30 ms, flip angle = 90°, 96 × 94 matrix, field of view = 240 × 40 mm^2^, voxel size = 2.5 × 2.5 × 3 mm^3^. For the resting-state sessions (before placebo administration, before and after the tasks), 355 volumes of T2^∗^- weighted echo-planar images (EPIs) were acquired for each session with the same parameters. All subjects were instructed to keep their eyes closed, to not think of anything specific, and to not fall asleep during the resting-state measurements.

### fMRI Preprocessing

Rs-fMRI-data were analyzed in MATLAB 2017 (The Mathworks Inc., Natick, MA, United States) using the SPM12 (Wellcome Department of Imaging Neuroscience, London, United Kingdom)^1^ and CONN toolboxes (Whitfield-Gabrieli and Nieto-Castanon, 2012). Preprocessing of the rs-fMRI data was performed using the adapted preprocessing pipeline in CONN. The pipeline includes motion correction (realignment and unwarping), slice-timing correction, automatic detection of artifactual scans (ART-based scrubbing, Mazaika et al., 2005), and normalization to MNI space. The CONN toolbox-featured intermediate scrubbing parameters were used to compute head motion in each session of each subject. In the next step, we evaluated the sessions in which head motion exceeded the threshold in more than 25% of volumes. This head motion criterion resulted in the exclusion of one subject’s RS0 and one subject’s RS2 session. Single-subject linear regression analyses were performed to remove effects of head motion (12 total motion covariates: six motion parameters plus temporal derivatives), physiological artifacts [10 total eigenvariates based on the anatomical component-based noise correction method (aCompCor, Chai et al., 2012): five each from eroded white matter (WM) and cerebrospinal fluid (CSF) masks], and artifactual scans in each subject during denoising in CONN. The resulting residual BOLD time series were band-pass filtered (0.01–0.25 Hz) and spatially smoothed with a 6 mm Full-Width at Half-Maximum (FWHM) Gaussian kernel. Finally, each time series was normalized to zero mean and unit variance (*z*-value), to reduce variance of non-neural origin (Huang et al., 2018). One single subject was not preprocessed due to the corruption of the data, therefore, in total 38 subjects (37 for each session) were included in the analysis.

### FC Between Network-Pairs

**FIGURE 2:**
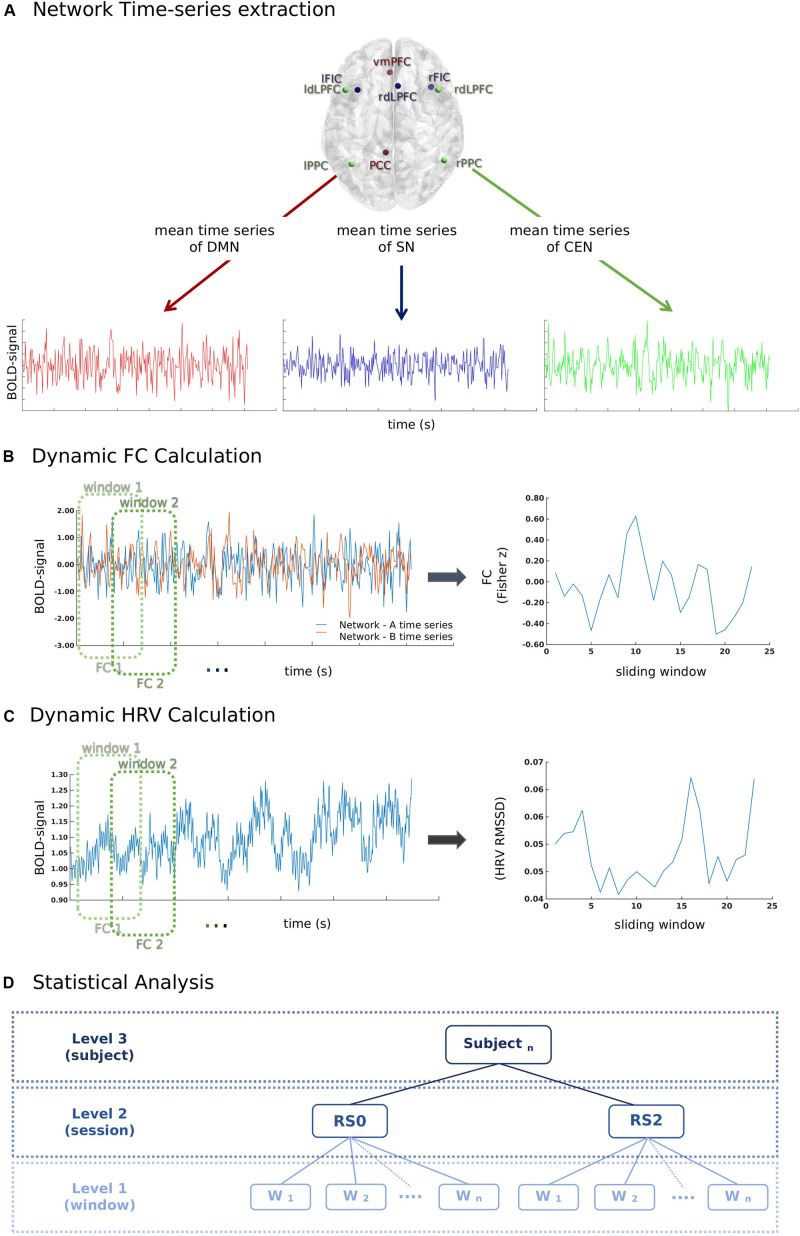
A schematic illustration of the methods used for the calculation of the temporal relationship between heart rate variability (HRV) and functional connectivity (FC) between network-pairs. **(A)** First, time series of mean BOLD signals in each network were extracted for each window (60 s) according to the sliding-window analysis approach. **(B)** Pearson-correlation was performed for the calculation of FC between network-pairs. **(C)** RMSSD was calculated from the inter-beat interval time series for each window (60 s) according to the sliding-window analysis approach. **(D)** The relationship between window-by-window RMSSD and FC between network-pairs was analyzed using a multilevel linear mixed model.

Key regions of three canonical brain networks were identified following prior literature (Uddin et al., 2011). As shown in Table 1, the resulting 10 region of interests (ROIs) were constructed by drawing spheres of 5 mm radius around the following key nodes: the DMN (vmPFC and PCC), SN [bilateral fronto-insular cortex (FIC) and rostral ACC (rACC)], and CEN (bilateral dlPFC and PCC) (Figure 2A).

**TABLE 1:** Composition of the canonical networks.

Network	Region	MNI coordinates (mm)
SN	rFIC	39, 23, −4
	lFIC	−34, 20, −8
	ACC	6, 24, 32
CEN	rDLPFC	46, 20, 44
	lDLPFC	−46, 20, 44
	rPPC	52, −52, 50
	lPPC	−40, −56, 44
DMN	vmPFC	−2, 38, −12
	PCC	−6, −44, 34

*SN, salience network; DMN, default mode network; CEN, central executive network; rFIC, right fronto-insular cortex; lFIC, left fronto-insular cortex; rDLPFC, right dorsal lateral prefrontal cortex; lDLPFC, left dorsal lateral prefrontal cortex; rPPC, right posterior parietal cortex; lPPC, left posterior parietal cortex; VMPFC, ventrolateral prefrontal cortex; PCC, posterior cingulate cortex.*

A sliding-window approach was used to calculate the FC between network-pairs (Figure 2B). The mean time course of each brain network was extracted for the windows of 60 s with a 50% overlap. The total number of windows for each session was 23. Pearson-correlation was calculated for the FC between network-pairs.

### Physiological Recordings and HRV Calculation

A finger PPG signal with a sampling rate of 500 Hz was acquired using the scanner’s built-in equipment concurrently with fMRI. During data acquisition, local maxima of PPG signal were automatically detected and timestamps of PPG peaks (P-peaks) were recorded by the MR scanner. The inter-beat intervals (IBIs) were calculated by extracting the time interval between subsequent P-peaks. Prior to the calculation of the inter-beat intervals, the quality of the peaks was manually inspected using an in-house MATLAB script by overlapping scanner detected P-peaks timestamps over PPG signal; missing peaks were added manually. The intermittent errors in P-peaks due to the ectopic beats or movement artifact were identified using percentage filter (IBIs increase or decrease of more than 30% compared to the previous interval) and subsequently interpolated (Salo et al., 2001; Ramshur, 2010; Peltola, 2012; Choi and Shin, 2018). RMSSD is the root mean square of the successive difference in adjacent IBIs, which measures the short-term variations of the IBI signal (Task Force of ESC and NASPE, 1996; Shaffer and Ginsberg, 2017). RMSSD was calculated for each individual sliding window of 60 s (Figure 2C).

### Statistical Analyses

The data of the current study was comprised of two resting-state sessions and 23 sliding window estimations for each subject. To assess dynamical temporal associations, the multilevel analysis was preferred over a summary-statistics model. A multilevel mixed linear model (Figure 2D) was build using the fitlme command of Statistics Toolbox in MATLAB. Window-by-window FC between network-pairs was taken as the dependent variable, while session (RS0 and RS2), network-pair (DMN-CEN, DMN-SN, and SN-CEN) and window-by-window HRV were added as regressors. The HRV values were centered according to the subject level mean HRV values for each session separately (Finch, n.d.). Fixed effects were calculated for the regressors and their interaction terms. The random term was defined as network-pairs nested under sessions and subjects. The random intercept model with a diagonal covariance matrix was chosen based on the Akaike Information Criterion (AIC) fit index. Parameters were estimated using the restricted maximum likelihood estimation (REML) method, which has been proven to be more accurate than maximum likelihood estimation (MLE) for estimating variance parameters (Kreft and De Leeuw, 1998; Finch, n.d.). The normality of residuals and homoscedasticity were investigated by plotting the normal probability of residuals and of residuals vs. fitted values (Supplementary Figure S1). Bonferroni adjustment was used for multiple comparison correction in *post-hoc* analyses (in total six pair-wise comparisons across the regression coefficients of each network-pair in each session). Mean HRV and mean HR in each session were also added separately to the above-specified model as second-level variables. To investigate the effect of acute stress induction on the temporal association of dFC between network-pairs, the parameters related to stress response, such as the difference between saliva alpha-amylase and cortisol levels before and after stress induction, and the difference between HR and ratings of VAS-Nerv, were added to the model on subject level. Age and period of placebo intake (Day 1 or Day 2) were also controlled for.

The effect of acute stress induction on mean HR was examined by one-way rmANOVA (Session: RS0, RS1, ScanSTRESS task and RS2). Greenhouse-Geisser was used to adjust the degrees of freedom for the averaged tests of significance. Additionally, the carry-over effect of stress induction on subjective and physiological measures was analyzed by conducting paired sample *t*-tests between RS0 and RS2, using mean HR and mean HRV during scans, saliva cortisol, and alpha-amylase levels, and scores of STAI-XI and VAS-Nerv at the time of each scanning time point as dependent variables. The statistical threshold was set to α = 0.05.

## Results

### The Carry-Over Effect of Acute Stress Induction on Subjective and Physiological Measures

**FIGURE 3:**
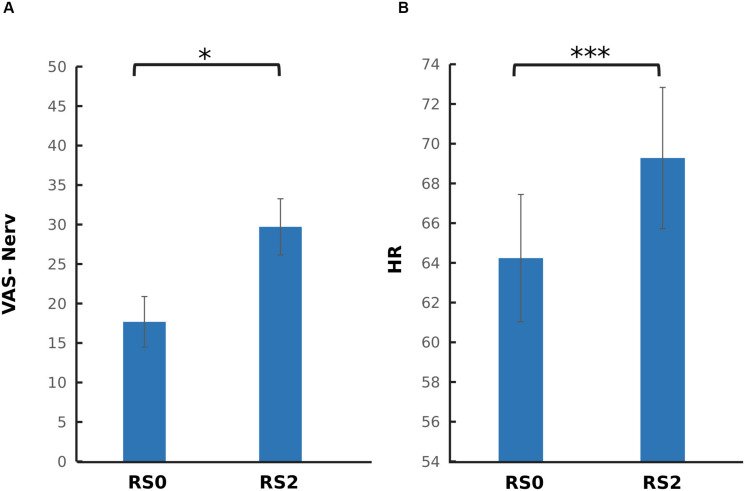
Comparison of psychophysiological parameters between the two sessions. **(A)** Visual analog scale for nervousness (VAS-Nerv) **(B)** Mean heart rate (HR). After the acute stress induction, a significant increase in the level of nervousness, and mean HR was found. The error bars represent standard error. ^∗^*p* < 0.05; ^∗∗∗^*p* < 0.001.

To investigate the carry-over effect of acute stress induction on subjective and physiological measures, paired sample *t*-tests were performed across RS0 and RS2. As depicted in Figure 3, a significant increase in nervouseness [*t*(37) = −2.44, *p* = 0.019] and HR [*t*(37) = −5.78, *p* < 0.001] were observed. No significant change was observed on the level of salivary cortisol and alpha-amylase, and mean RMSSD.

### The Dynamic Temporal Association of HRV With FC Between Network-Pairs

**FIGURE 4:**
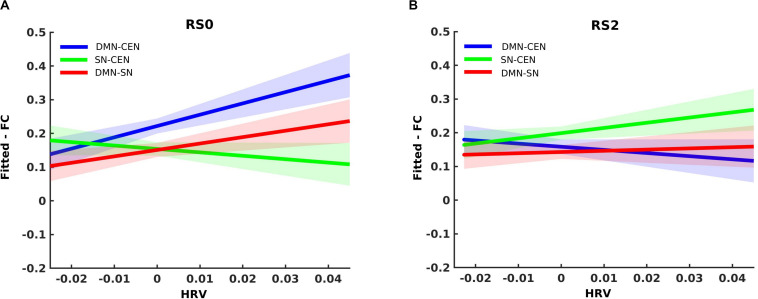
Differential temporal association between heart rate variability (HRV) and functional connectivity (FC) between network-pairs across sessions (RS0 and RS2). **(A)** The multilevel linear mixed effect model showed a significant correlation between HRV and FC between DMN-SN, and DMN-CEN during baseline (RS0). The strength of the association between HRV and FC was significantly stronger for DMN-CEN than for SN-CEN during baseline session (RS0). **(B)** The correlation between HRV and FC between DMN-CEN was significantly weaker during the second session (RS2) in comparison to baseline. RS0, Baseline Session; RS2, After stress induction; SN, Salience Network; DMN, Default Node Network; CEN, Central Executive Network. Shaded areas indicate standard error.

Analysis using a multilevel linear mixed model was conducted by taking window-by-window FC (dFC) between network-pairs as the dependent variable and window-by-window HRV, network-pairs and session as regressors, while subject level random terms for each session were introduced. HRV was significantly associated with dFC between DMN-CEN [*b* = 3.35, *t*(5025) = 3.47, *p* < 0.006 (Bonferroni corrected)] in the baseline condition (Table 2). As depicted in Figure 4A, this association was significantly higher than that between SN-CEN dFC and HRV [*b* = −4.36, *t*(5025) = −3.20, *p* < 0.006 (Bonferroni corrected)] at baseline (Table 3).

**TABLE 2:** Correlation of RMSSD with dFC between network-pairs for each session (RS0 and RS2).

Session	FC	Estimate	SE	T	Adjusted *P*-value
RS0	DMN-SN	1.90	0.96	1.97	0.288
	DMN-CEN	3.35	0.96	3.47	0.003**
	SN-CEN	–1.01	0.96	–1.05	1.000
RS2	DMN-SN	0.36	0.94	0.38	1.000
	DMN-CEN	–0.93	0.94	–0.99	1.914
	SN-CEN	1.53	0.93	1.64	0.600

RMSSD = Root mean square of the successive difference in adjacent IBIs; dFC, dynamic functional connectivity; RS0, Baseline session; RS2, After stress induction; SN, Salience network; DMN, Default mode network; CEN, Central executive network; Adjusted p-value, Bonferroni corrected p-values. Bold font represents significant results; ** represents p < 0.01.

**TABLE 3:** Within-session comparisons of correlation strengths between RMSSD and network-pairs dFC.

Session	Reference NP	Target NP	Estimate	SE	T	Adjusted *P*-value
RS0	DMN-SN	SN-CEN	–2.92	1.36	–2.13	0.192
	DMN-CEN	DMN-SN	–1.45	1.36	–1.06	1.000
	DMN-CEN	SN-CEN	–4.36	1.36	–3.20	0.006**
RS2	DMN-SN	SN-CEN	1.18	1.32	0.89	1.000
	DMN-CEN	DMN-SN	1.28	1.32	0.97	1.000
	DMN-CEN	SN-CEN	2.47	1.32	1.87	0.372

RMSSD, Root mean square of the successive difference in adjacent IBIs; dFC, dynamic functional connectivity; NP, Network-pair; RS0, Baseline session; RS2, After stress induction; SN, Salience network; DMN, Default mode network; CEN, Central executive network; Adjusted P-value, Bonferroni corrected p-values. Bold font represents significant results; **p < 0.01.

The relationship between HRV and DMN-CEN-dFC significantly decreased after acute stress induction [*b* = −3.35, *t*(5025) = −3.47, *p* < 0.006 (Bonferroni corrected)] (Figure 4B and Table 4) and was not significant at the RS2.

**TABLE 4:** Between-session comparisons of correlation strengths between RMSSD and network-pairs dFC.

NP	Reference session	Target session	Estimate	SE	T	Adjusted *P*-value
DMN-SN	RS0	RS2	–1.54	1.34	–1.23	1.000
DMN-CEN	RS0	RS2	–4.28	1.34	–3.18	0.006**
SN-CEN	RS0	RS2	2.55	1.34	1.90	0.342

RMSSD, Root mean square of the successive difference in adjacent IBIs; dFC, dynamic functional connectivity; NP, Network-pair; RS0, Baseline session; RS2, After stress induction; SN, Salience network; DMN, Default mode network; CEN, Central executive network; Adjusted P-value, Bonferroni corrected p-values. Bold font represents significant results; **p < 0.01.

These effects were also observed after controlling for age and the period of placebo intake, as well as mean HR and mean HRV during each session. Moreover, additional analyses including the difference between saliva alpha-amylase and cortisol levels, and the difference between ratings of VAS-Nerv parameters before and after stress induction did not alter the above-mentioned findings.

## Discussion

In this study, we examined the association of RMSSD with dFC between brain networks at baseline and right after a psychosocial stress task in a sample of 38 healthy male subjects. By using multilevel regression analysis, we demonstrated that the minute-by-minute association between the dFC and RMSSD was significantly stronger for the interaction between DMN and CEN than between SN and CEN in the baseline session. This difference between dFC of network-pairs in terms of their association with RMSSD disappeared after the stress task. Furthermore, dFC between DMN and CEN showed a weaker correlation with RMSSD after the stress task in comparison to baseline. Moreover, these findings were replicated with one additional resting-state scan (RS1), which was acquired after placebo intake and before the stress induction (Supplementary Results). These results indicate a dynamic functional relationship between HRV and brain networks that varies depending on external conditions. To the authors’ knowledge, this is the first report suggesting the pattern of functional associations between HRV and the interaction between different brain networks during resting state, and how this changes immediately after acute stress induction.

The significant correlation between RMSSD and dFC of DMN and CEN in the baseline session suggests that the CEN nodes, such as dlPFC and posterior parietal cortex (PPC), are functionally related to the rhythmic activity of the heart, even though they are not part of the original definition of CAN (Benarroch, 1993). The stronger functional association of RMSSD with the interaction between CEN and DMN compared with CEN and SN during the baseline session is in line with the previously mentioned extended NVI model, in which the role of CEN in vagal control was proposed (Smith et al., 2017). This model describes that DMN plays a role in the conceptualization of the visceral and somatic information, thereby representing the conceptual significance of the overall situation of the organism in a given context, while SN plays a regulatory role at the level of perception (Barrett and Satpute, 2013; Smith et al., 2017). The synchronous activity of SN and DMN with CEN enables maintenance and further processing of relevant information (Dehaene, 2014; Barrett, 2016; Smith et al., 2017). Activation and FC of DMN regions are associated with spontaneous and self-generated thoughts under resting conditions (Raichle et al., 2001; Buckner et al., 2008; Buckner and DiNicola, 2019). On the other hand, SN activation and FC are induced in response to salient and affective stimuli and play a role in the allocation of attention (Seeley et al., 2007; Hermans et al., 2011, 2014; Menon, 2011). Therefore, under resting conditions, the dominance of DMN was expected. In this study, the static rsFC between CEN and DMN is stronger than between CEN and SN at baseline (Supplementary Results). Presumably, HRV is primarily related to the currently dominant process across brain networks, which is expected to mirror the needs of the body, while on the other hand, HRV also reflects the processes in the brain invoked in the frame of its relation to the body and environment (McCraty and Childre, 2010).

Acute stress induction can induce a reorganization of FC between brain networks to support a hypervigilant state (Hermans et al., 2014). Since the stress response continues directly after stress exposure (van Marle et al., 2010), a carry-over effect of the stress task was expected during the following resting-state scan. Indeed, even the effects of prior cognitive tasks are carried over to the following post-task resting-state brain activity (Albert et al., 2009; Lewis et al., 2009; Hartzell et al., 2015). In this study, the association between HRV and dFC between CEN and DMN was decreased directly after acute stress induction when compared with the baseline session. An increase in FC and activation of SN regions was reported after acute stress induction, while DMN FC was decreased (van Marle et al., 2010; Vaisvaser et al., 2013; Quaedflieg et al., 2015; van Oort et al., 2017). The static rsFC findings of this study showed that rsFC between DMN-CEN was reduced after acute stress induction, which might explain the shift in the association of RMSSD with FC between network-pair. Of note, the carry-over effect of stress induction was observed regardless of the inclusion of mean values of HR, HRV, or nervousness ratings during each session. Participants reacted to the acute stress induction with an increase in HR during the task, which also did not explain the carry-over effect of stress on the association between HRV and interaction between intrinsic networks.

A point that needs to be taken into consideration is the usage of Pearson correlation coefficient to estimate the strength of FC between network-pairs. The Pearson correlation coefficient does not imply causal relationship and might also reflect an indirect influence by a third region (Wang et al., 2016). Therefore, current findings cannot indicate the direction of an effect between RMSSD and intrinsic brain networks. Even though HRV has been mostly interpreted as an index of the modulatory effect of the CAN on the cardiac activity, the CAN receives continuous information from the cardiovascular system via the cardiac afferent fibers and through blood vessels. Therefore, it is difficult to identify causality for the heart-brain circuit. For this reason, we chose to describe our observations as co-evolution of signals instead of an effect of one on the other. Likewise, we avoided limiting the association of RMSSD and functional association of brain networks in a frame of parasympathetic activity. RMSSD reflects predominantly parasympathetic rather than sympathetic activity (Shaffer and Ginsberg, 2017). However, confidently separating sympathetic and parasympathetic influences on the heart-brain circuit is beyond this study due to the complexity of the heart-brain circuit, which also involves interaction between the branches of the autonomic nervous system (ANS) and the non-linear interplay of all regulatory loops involving the intracardiac nervous system and pacemaker cells in the heart, especially when some of our ROIs are considered, which are not primarily part of the CAN. Moreover, additional analyses with other vagal HRV metrics, such as high-frequency HRV, are required to indicate that the functional association between the core brain regions and RMSSD was driven by parasympathetic activity.

A limitation of the current study is the window size of the 60 s, which might not be the optimal time window to capture the temporal dynamics of the interplay between RMSSD and intrinsic brain networks. While the underlying temporal scale of the reported effects has not been exhaustively assessed, the 60 s window size was used as a compromise between including enough timepoints within a window to provide reliable measures and capturing relatively short-lived effects (Hutchison et al., 2013; Chen et al., 2017). In the current study, the selection of a 60 s window and 50% overlap yielded 23 sliding-window measurements per scan, 30 fMRI timepoints per window for estimating FC and the association between dFC and RMSSD. According to previous findings in the literature, functionally relevant dFC patterns can be isolated from a window size of 60 s (Shirer et al., 2012; Gonzalez-Castillo et al., 2015; Leonardi and Van De Ville, 2015; Liégeois et al., 2017). Furthermore, the calculation of RMSSD from the signal acquired by means of recordings of less than 1 min is still a matter of debate (Laborde et al., 2017). This also corresponds to one of the reasons behind our choice of RMSSD as an HRV index parameter.

Since ECG is more susceptible to radiofrequency artifacts during fMRI scans and the magnetic field within the scanner, the pulse oximetry is generally preferred in MRI settings (Chang et al., 2013; Bellot et al., 2016; Kasper et al., 2017). Even though the utility of the pulse oximeter in calculating HRV by peak detection algorithms was demonstrated (Chang and Glover, 2009; Verstynen and Deshpande, 2011; Nilsson, 2013; Schäfer and Vagedes, 2013; Caballero-Gaudes and Reynolds, 2017), pulse oximeter measurements are delayed due to the pulse transit time. However, in MRI settings pulse oximetry is still more favorable than ECG because of its robustness to artifacts arising from the setting itself.

The HRV signal has a complex structure and involves superimposed oscillations (Ivanov et al., 1999; Pikkujämsä et al., 1999). RMSSD is one of the most commonly used time-domain measures of HRV; however, there are many other options to calculate HRV, such as time-domain, frequency-domain, and non-linear measurements (Shaffer and Ginsberg, 2017). Thus, RMSSD parameter illuminates only a specific and small part of HRV and cannot be considered a full representative of this regulation. Because of the 60 s window size, the calculation of the low-frequency domains is not appropriate from a signal analysis perspective (Task Force of ESC and NASPE, 1996; Shaffer and Ginsberg, 2017). Moreover, the frequency domain and also other time-domain indices are more susceptible to the influence of respiration, which was not controlled for in HRV calculation because neither respiratory rate nor depth was recorded during the scanning. Of note, the validity of ultra-short HRV features (acquisition time less than 5 min) is still under debate (Shaffer and Ginsberg, 2017; Castaldo et al., 2019); however, the use of acquisition times of 60 s and below was also proposed (Salahuddin et al., 2007; Esco and Flatt, 2014; Baek et al., 2015). On the other hand, the conventional recording time of 5 min would not be appropriate to examine the dynamic temporal functional association between the rhythmic activity of the heart and the FC of the core brain networks.

Physiological signals are generally regressed out during pre-processing of fMRI data as respiration and the cardiac cycle can result in neuronal and non-neuronal fluctuations in the BOLD signal due to the systemic changes in arterial CO2 concentrations and blood flow (Birn et al., 2006; Shmueli et al., 2007; Chang and Glover, 2009). In the current study, a CompCor approach was used during pre-processing that extracts multiple nuisance regressors from the voxels within WM and CSF via principal component analysis (Behzadi et al., 2007; Muschelli et al., 2014). A CompCor approach can account for physiological noise (Behzadi et al., 2007) and head motion (Muschelli et al., 2014). As our main interest was the interaction of cardiac activity and inter-network FC, removing the physiological signals from the imaging data further than regressing out the signal intensity from WM and CSF might have resulted in a decrease of the signal of interest (Khalili-Mahani et al., 2013; Zhang et al., 2019). Nevertheless, considering the parallel change between the results of static rsFC between the network-pairs (Supplementary Results) and the dynamic association of HRV and dFC between network-pairs, the findings of this study should be interpreted with caution.

These findings, although preliminary, suggest that HRV co-fluctuates with the core brain networks selectively depending on the condition. This combination of findings provides some support for the conceptual premise that the brain and the heart function in a closely coordinated manner as a part of a bigger psychophysiological system to maintain the homeostatic state of the organism in a constantly changing environment.

## Data Availability Statement

The datasets generated for this study are available on request to the corresponding author.

## Ethics Statement

The studies involving human participants were reviewed and approved by the Ethics Committee of the Medical Faculty of the University of Magdeburg. The patients/participants provided their written informed consent to participate in this study.

## Author Contributions

TC, ZS, SA, LH, HJ, and MW were involved in the development of the study. TC, ML, GW, AL, LD, MW, and ZS were involved in the analysis and interpretation of data. All authors gave approval of the final version and agreed to be accountable for all aspects of the work.

## Conflict of Interest

MW received institutional research support from Heel paid to his institution for this study and from BrainWaveBank and H. Lundbeck A/S outside the submitted work. The University of Tübingen received institutional fees for advisory services by MW from Heel GmbH, Servier Deutschland GmbH, Bayer AG, and Janssen-Cilag GmbH. HJ was supported by the fortüne grant of the Medical Faculty of the University of Tübingen (No. 2487-1-0). TC, ML, LH, LD, and ZS received financial support paid to the institurion for conference attendance from Heel outside the submitted work. The remaining authors declare that the research was conducted in the absence of any commercial or financial relationships that could be construed as a potential conflict of interest.

## Footnotes

1 http://www.fil.ion.ucl.ac.uk
