# Supplementary material for: Heart Rate Variability as an Index of Differential Brain Dynamics at Rest and After Acute Stress Induction
Source: Front Neurosci. 2020 Jul 2;14:645. doi: 10.3389/fnins.2020.00645 (PMC7344021; doi:10.3389/fnins.2020.00645)
Supplement: Supplementary file 1 [file Table_1.DOCX]

Supplementary Material

1. Normality plots of the multilevel linear mixed model

**Figure S1:** Residual analysis of the multilevel mixed model conducte
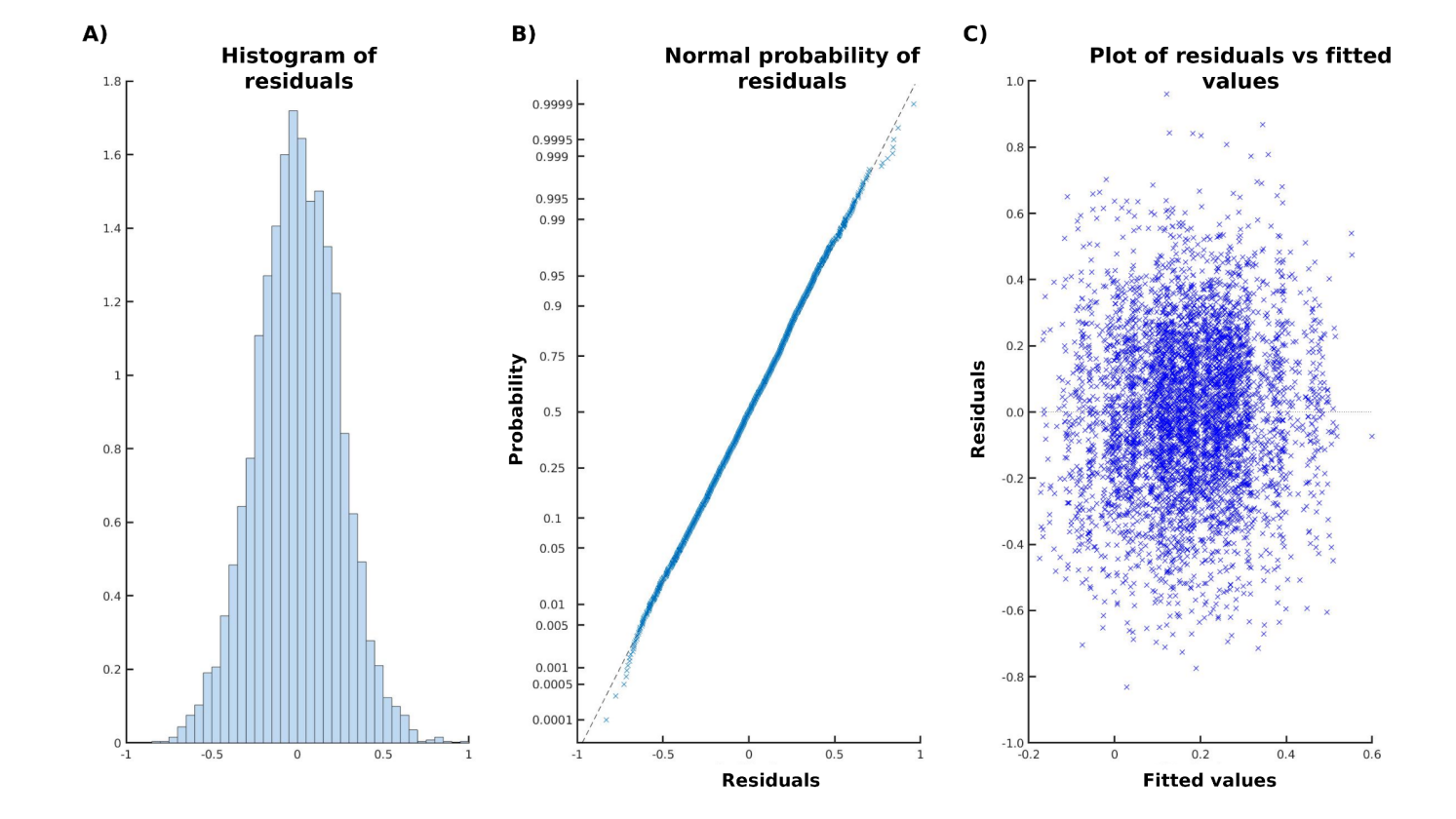
d for the temporal association between heart rate variability and dynamic functional connectivity. **A)** Histogram of residuals. **B)** Test for normal probability of residuals. **C)** Scatterplot of residuals vs. fitted values.

1. Effect of acute stress induction on heart rate

The effect of acute stress induction on mean HR was tested using one-way rmANOVA (Session: RS0, RS1, adapted ScanSTRESS task and RS2). A significant effect of session was found (F (1.37, 50.93) = 113.77, p < 0.001). HR was significantly higher during the stress task than during RS0 (MD = 20.07 [95% CI = 16.30 – 23.83], p < 0.001), RS1 (MD = 19.44 [95% CI = 16.23 – 22.66], p < 0.001) and RS2 (MD = 15.03 [95% CI = 12.84–17.22], p < 0.001). HR was also higher during RS2 than during RS0 (MD = 5.03 [95% CI = 3.06 – 7.00], p < 0.001) and RS1 (MD = 4.41 [95% CI = 2.82 – 6.00], p < 0.001) (Figure S2).


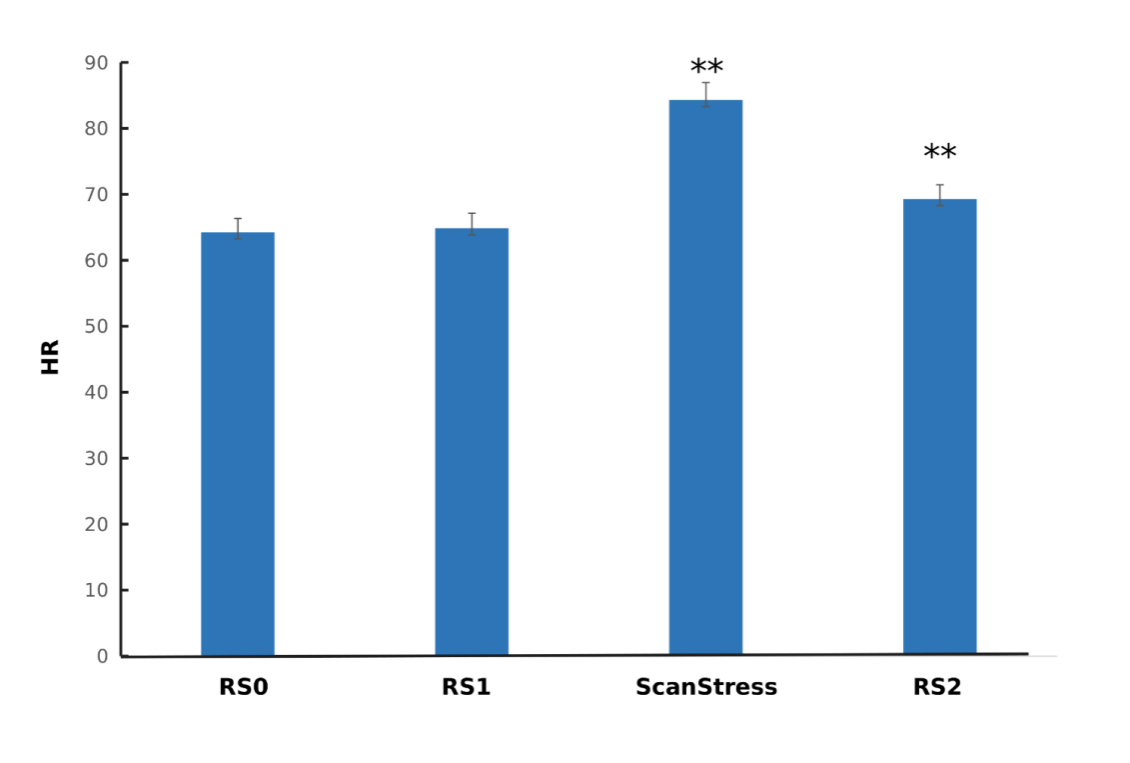


**Figure S2:** Effect of acute stress induction on heart rate (HR). HR was significantly higher during the stress task (adapted version of the ScanStress) compared to RS0 (MD= 20.07 [95% CI 16.30 – 23.83], *p* <0.001), RS1 (MD= 19.44 [95% CI 16.23 – 22.66], *p* < 0.001) and RS2 (MD= 15.03 [95% CI 12.84–17.22], *p* < 0.001). A significant increase in HR was also observed in RS2 compared to RS0 (MD= 5.03 [95% CI 3.06–7.00], *p* < 0.001) and RS1 (MD= 4.41 [95% CI 2.82–6.00], *p* < 0.001) condition. (RS0 = Baseline Session; RS1 = resting period between placebo and stress induction; ScanStress = During the ScanStress task; RS2 = after stress induction.).

1. The Carry-over Effect of Acute Stress Induction on resting-state functional connectivity

The carry-over effect of acute stress induction on resting-state functional connectivity (rsFC) was examined by a linear mixed model using the fitlme command in MATLAB. FC between network-pairs during the whole scan was used as the depended variable and session (RS0 and RS2), network-pair (DMN-SN, DMN-CEN, and SN-CEN) and session x network-pair were used as fixed effect terms, while subject was taken as random term. The full covariance matrix was chosen and the parameters were estimated by REML.

In baseline (RS0), rsFC between DMN-CEN was significantly stronger compared to both DMN-SN (t (210) = -2.47, *p* = 0.01) and SN-CEN (t (210) = -2.26, *p*= 0.02). After acute stress induction, rsFC between DMN-CEN (t (210) = -1.97, *p* = 0.05) was significantly reduced (Figure S3).


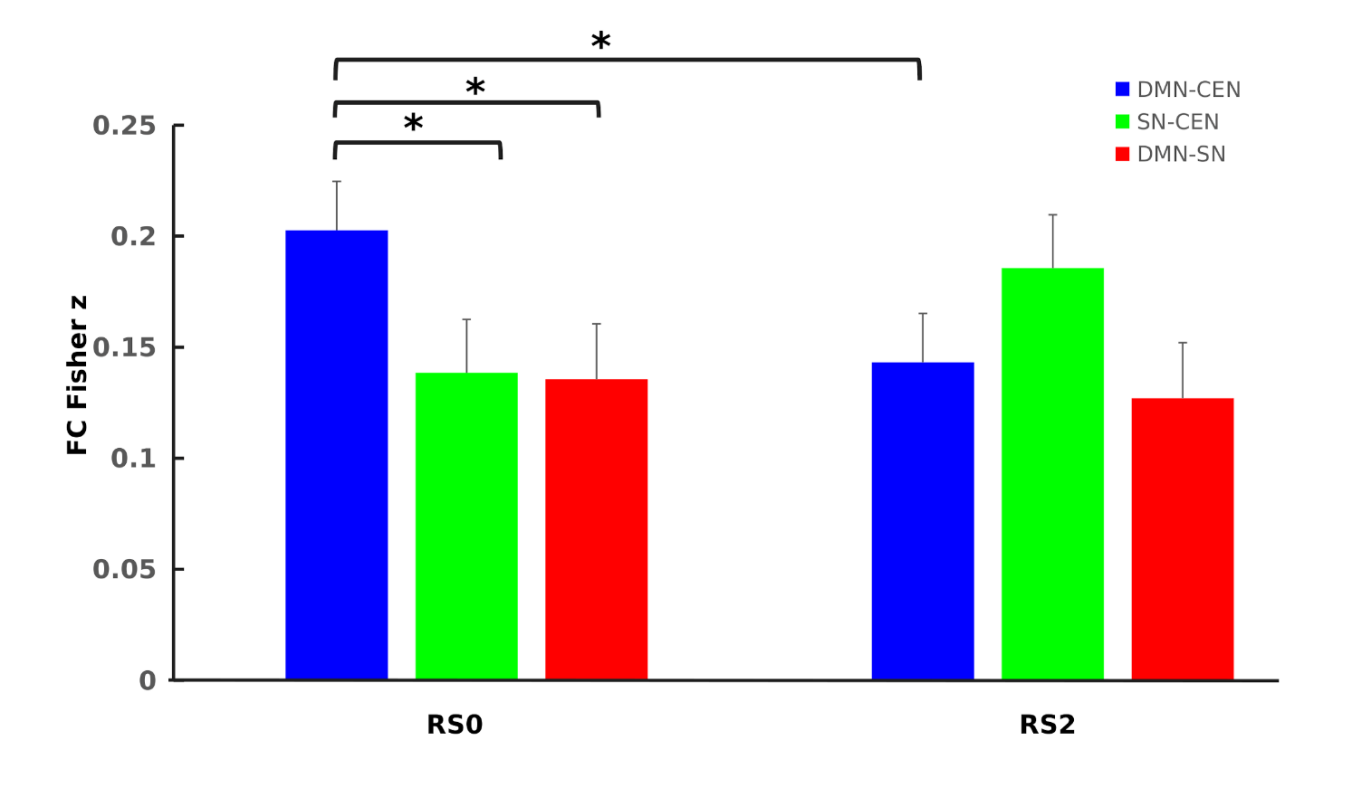


**Figure S3:** Resting-state functional connectivity (rsFC) between DMN-CEN, SN-CEN and DMN-SN at RS0 and RS**2**. In RS0, DMN-CEN showed a significantly stronger rsFC than DMN-SN and SN-CEN pairs. DMN-CEN rsFC was significantly reduced in RS2 as compared to RS0. (RS0 = Baseline Session; RS2 = After stress induction; SN = Salience Network; DMN = Default Node Network; CEN = Central Executive Network; * *p* < 0.05).

1. Dynamic temporal association between heart rate variability and dynamic functional connectivity between Network-pairs in RS1

This study included three resting state functional magnetic resonance imaging scans. The first one was acquired at baseline (RS0), the second one (RS1) was acquired after placebo intake and just before the ScanSTRESS task and the third one (RS') was acquired immediately after the ScanSTRESS task. Because placebo intake can induce changes in resting state brain activity (Tétreault et al., 2016; Wager et al., 2004), we restricted our main analyses to the comparison of RS0 and RS2. To explore the dynamic temporal association between HRV and dFC between network-pairs in RS1, additional analyses were built using a multilevel linear mixed model with a diagonal covariance matrix, where window-by-window FC between network-pairs was used as the dependent variable, while session (RS0, RS1 and RS2), network-pair (DMN-CEN, DMN-SN, and SN-CEN) and window-by-window HRV were added as regressors in parallel to the main analyses in the manuscript.

Comparable to the RS0 findings, also in RS1 window-by-window HRV was correlated to dFC between DMN-SN (b = 3.78, t(7641) = 4.45, *p* < 0.001 (uncorrected) and DMN-CEN (b = 2.86, t(7641) = 3.37, *p* < 0.001 (uncorrected) FC with HRV in RS1 (Table S1). As depicted in Figure S4, the temporal association of SN-CEN dFC with HRV was significantly weaker than the association of DMN-SN dFC (b = -3.48, t(7641) = - 2.93, *p* < 0.003 (uncorrected) and DMN-CEN dFC with HRV (b = -2.68, t(7641) = -2.23, *p* < 0.02 (uncorrected) in RS1 as it was found in RS0 (Table S2).

After acute stress induction the association of HRV with DMN-CEN dFC (b= -3.78, t (7641) = -3.002, *p* = 0.003 (uncorrected)) was significantly weaker in comparison to RS1 as it was in comparison to RS0. In addition, the association between HRV and DMN-SN dFC (b = -3.42, t (7641) = -2.72, p= 0.006 (uncorrected)) was significantly decreased in RS1 in comparison to RS2 (Table S3).

There were no significant differences between RS0 and RS1 in terms of differential association of HRV and dFC between network-pairs (Table S3). These findings were also observed after controlling for the effect of age and the order of placebo intake.

**Figure S4:** Differential temporal association between heart rate variability (HRV) and functional
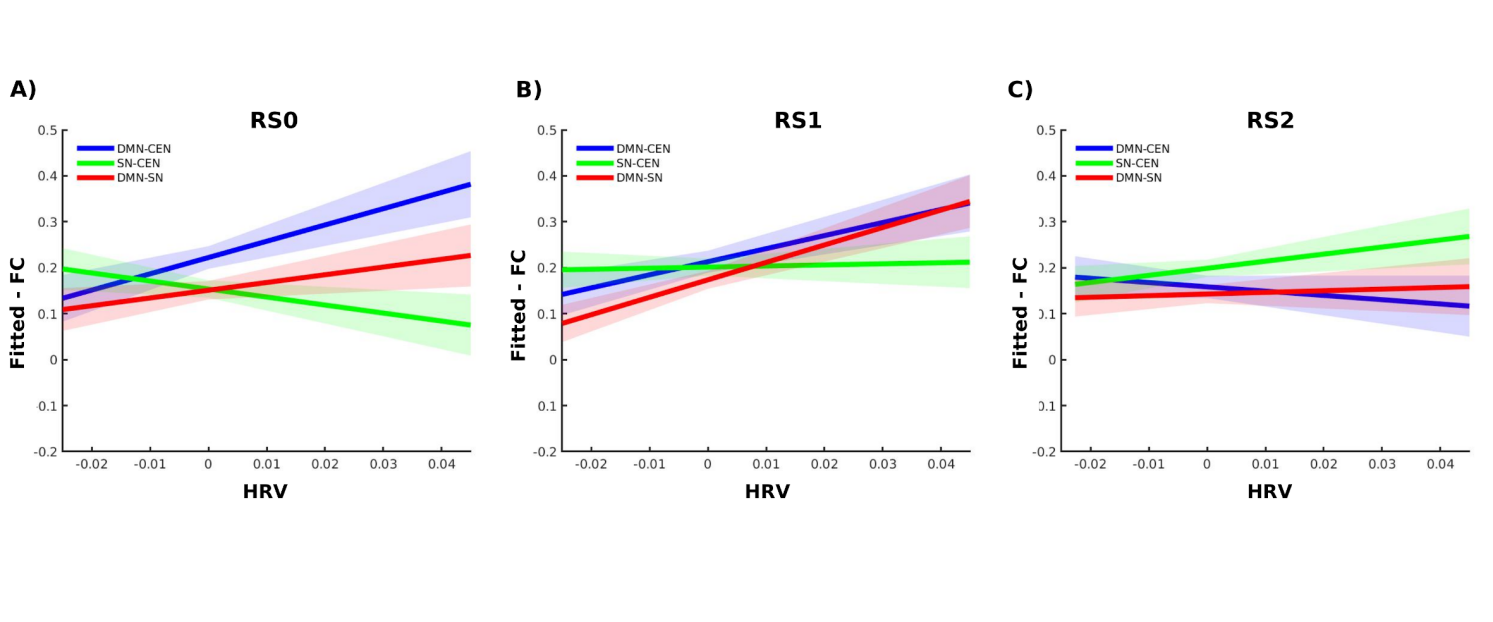
 connectivity (FC) between network-pairs across three sessions (RS0, RS1 and RS2). **A)** The multilevel linear mixed effect model showed a significant correlation between HRV and FC between DMN-CEN at baseline (RS0). The strength of association between HRV and dFC was significantly stronger for DMN-CEN than for SN-CEN in the baseline session (RS0). **B)** Pearson correlation between HRV and dFC DMN-SN and dFC DMN-CEN in RS1. The association between HRV and dFC was significantly stronger for DMN-CEN than for SN-CEN in RS1. **C)** The correlation between HRV and FC between DMN-CEN was significantly weaker in the second session (RS2) in comparison to RS0 and RS1. (RS0 = Baseline Session; RS1 = resting period between placebo and stress induction; RS2 = after stress induction; SN = Salience Network; DMN = Default Node Network; CEN = Central Executive Network; Shaded areas indicate standard error.).

**Table S1:** Association of heart rate variability with dynamic functional connectivity between network-pairs for each session (RS0, RS1 and RS2)

| **Session** | **FC** | **Estimate** | **SE** | **T** | **Unadjusted**  **P-value** | **Adjusted**  **P-value** |  |
| --- | --- | --- | --- | --- | --- | --- | --- |
|  | DMN-SN | 1.62 | 1.05 | 1.53 | 0.12 | 1.00 |  |
| **RS0** | DMN-CEN | 3.50 | 1.05 | 3.33 | **<0.001**** | **<0.01**** |  |
|  | SN-CEN | -1.77 | 1.05 | -1.68 | 0.092 | 0.82 |  |
| **RS1** | DMN-SN | 3.78 | 0.84 | 4.50 | **<0.001***** | **< 0.01**** |  |
|  | DMN-CEN | 2.86 | 0.85 | 3.37 | **<0.001***** | **< 0.01**** |  |
|  | SN-CEN | 0.26 | 0.84 | 0.31 | 0.759 | 1.00 |  |
| **RS2** | DMN-SN | 0.36 | 0.93 | 0.38 | 0.702 | 1.00 |  |
|  | DMN-CEN | -0.93 | 0.94 | -0.99 | 0.319 | 1.00 |  |
|  | SN-CEN | 1.54 | 0.93 | 1.65 | 0.09 | 0.81 |  |

Note: RS0 = Baseline Session; RS1= After placebo intake, before stress indcution; RS2 = After stress induction; SN = Salience Network; DMN = Default Node Network; CEN = Central Executive Network; Adjusted *p*-value= Bonferroni corrected *p-*values; Bold font represents significant results; * represents *p* < 0.05; ** represents *p* < 0.01.

**Table S2:** The within-session comparisons of correlation strengths between HRV and dFC between network-pairs

| **Session** | **Reference**  **NP** | **Target**  **NP** | **Estimate** | **SE** | **T** | **Unadjusted**  **P-value** | **Adjusted**  **P-value** |  |
| --- | --- | --- | --- | --- | --- | --- | --- | --- |
| **RS0** | DMN-SN | SN-CEN | -3.40 | 1.49 | -2.28 | **0.023*** | 0,18 |  |
|  | DMN-CEN | DMN-SN | -1.87 | 1.49 | -1.25 | 0.210 | 1.00 |  |
|  | DMN-CEN | SN-CEN | -5.28 | 1.49 | -3.54 | **<0.001***** | **<0.01**** |  |
| **RS1** | DMN-SN | SN-CEN | -3.48 | 1.19 | -2.93 | **0.003**** | **0.01**** |  |
|  | DMN-CEN | DMN-SN | 1.19 | 1.20 | 1.00 | 0.318 | 1.00 |  |
|  | DMN-CEN | SN-CEN | -2.68 | 1.20 | -2.23 | **0.025*** | 0.22 |  |
| **RS2** | DMN-SN | SN-CEN | 1.18 | 1.32 | 0.89 | 0.371 | 1.00 |  |
|  | DMN-CEN | DMN-SN | 1.28 | 1.32 | 0.97 | 0.330 | 1.00 |  |
|  | DMN-CEN | SN-CEN | 2.47 | 1.32 | 1.87 | 0.062 | 0.54 |  |

Note: NP = Network-pair; RS0 = Baseline Session; RS1= After placebo intake, before stress indcution; RS2 = After stress induction; SN = Salience Network; DMN = Default Node Network; CEN = Central Executive Network; Adjusted P-value= Bonferroni corrected *p* values; Bold font represents significant results; * represents p < 0.05; ** represents p < 0.01.

**Table S3:** The between-session comparisons of correlation strengths between HRV and network-pairs dFC

| **NP** | **Reference**  **Session** | **Target**  **Session** | **Estimate** | **SE** | **T** | **Unadjusted**  **P-value** | **Adjusted**  **P-value** |  |
| --- | --- | --- | --- | --- | --- | --- | --- | --- |
| DMN-SN | RS0 | RS1 | 2.17 | 1.35 | 1.61 | 107 | 0.96 |  |
|  | RS0 | RS2 | -1.26 | 1.41 | -0.90 | 0.369 | 1.00 |  |
|  | RS1 | RS2 | -3.43 | 1.25 | -2.73 | **0.006**** | **0.05*** |  |
| DMN-CEN | RS0 | RS1 | -0.65 | 1.35 | -0.48 | 0.631 | 1.00 |  |
|  | RS0 | RS2 | -4.44 | 1.41 | -3.15 | **<0.002**** | **0.01**** |  |
|  | RS1 | RS2 | -3.79 | 1.26 | -3.00 | **<0.003**** | **0.01**** |  |
| SN-CEN | RS0 | RS1 | 2.03 | 1.35 | 1.51 | 0.131 | 1.00 |  |
|  | RS0 | RS2 | 3.31 | 1.41 | 2.35 | **0.018*** | 0.16 |  |
|  | RS1 | RS2 | 1.28 | 1.26 | 1.02 | 0.308 | 1.00 |  |

Note: NP = Network-pair; RS0 = Baseline Session; RS1= After placebo intake, before stress indcution; RS2 = After stress induction; SN = Salience Network; DMN = Default Node Network; CEN = Central Executive Network; Adjusted P-value= Bonferroni corrected *p* values; Bold font represents significant results; * represents p < 0.05; ** represents p < 0.01.

1. **The distribution of the windowed time series for each network**

**
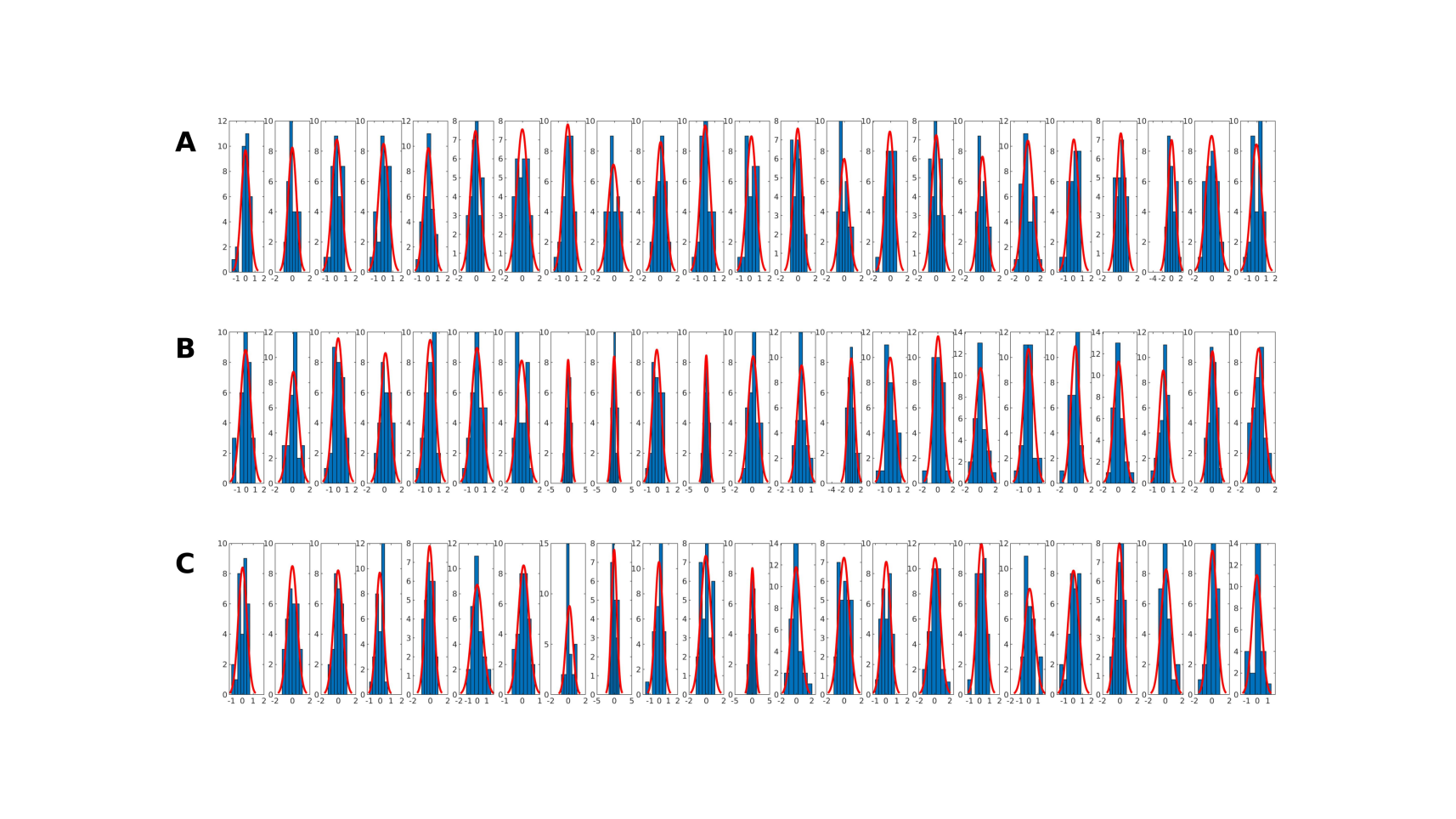
**

**Figure S5:** The distribution of the windowed time series for each network for a typical subject (P0010-ld98-010) of one resting-state session (RS0). Here each row represents each network (A). Default Mode Network (DMN), (b). Salience Network (SN), (c). Central Executive Network (CEN) and each column represents a window.

**References**

Wager, T. D., Rilling, J. K., Smith, E. E., Sokolik, A., Casey, K. L., Davidson, R. J., … Cohen, J. D. (2004). Placebo-Induced Changes in fMRI in the Anticipation and Experience of Pain. *Science*, *303*(5661), 1162–1167. https://doi.org/10.1126/science.1093065

Tétreault, P., Mansour, A., Vachon-Presseau, E., Schnitzer, T. J., Apkarian, A. V., & Baliki, M. N. (2016). Brain Connectivity Predicts Placebo Response across Chronic Pain Clinical Trials. PLOS Biology, 14(10), e1002570. https://doi.org/10.1371/journal.pbio.1002570
